# Study on prevention of hypercapnia by nasal high flow in patients undergoing endoscopic retrograde cholangiopancreatography during intravenous anesthesia

**DOI:** 10.1097/MD.0000000000020036

**Published:** 2020-05-08

**Authors:** Takao Ayuse, Hironori Sawase, Eisuke Ozawa, Kazuyoshi Nagata, Naohiro Komatsu, Takuro Sanuki, Shinji Kurata, Gaku Mishima, Naoki Hosogaya, Sawako Nakashima, Max Pinkham, Stanislav Tatkov, Nakao Kazuhiko

**Affiliations:** a Division of Clinical Physiology, Department of Translational Medical Sciences; b Department of Gastroenterology and Hepatology, Nagasaki University Graduate School of Biomedical Sciences; c Department of Dental Anesthesiology, Nagasaki University Hospital; d Nagasaki University Hospital, Clinical Research Center, Nagasaki, Japan; e Fisher & Paykel Healthcare Ltd, Auckland, New Zealand.

**Keywords:** hypercapnia, intravenous anesthesia, nasal high flow

## Abstract

**Background:**

For relatively invasive upper gastrointestinal endoscopy procedures, such as an endoscopic retrograde cholangiopancreatography (ERCP), and also lower gastrointestinal endoscopy procedures, intravenous anesthesia is routinely used to reduce patient anxiety. However, with the use of intravenous anesthesia, even at mild to moderate depth of anesthesia, there is always a risk of upper airway obstruction due to a relaxation of the upper airway muscles.

With the advent of nasal high flow (NHF) devices that allow humidified high flow air through the nasal cavity, can be used as a respiratory management method in the context of anesthesia. AIRVO is commonly used for patients with obstructive sleep apnea and other respiratory disorders. This device uses a mild positive pressure load (several cmH_2_O) that improves carbon dioxide (CO_2_) washout and reduces rebreathing to improve respiratory function and therefore is widely used to prevent hypoxemia and hypercapnia.

This study aims to maintain upper airway patency by applying NHF with air (AIRVO) as a respiratory management method during intravenous anesthesia for patients undergoing an ERCP. In addition, this study investigates whether the use of an NHF device in this context can prevent intraoperative hypercapnia and hypoxemia.

**Methods/design:**

This study design employed 2 groups of subjects. Both received intravenous anesthesia while undergoing an ERCP, and 1 group also used a concurrent nasal cannula NHF device. Here we examine if the use of an NHF device during intravenous anesthesia can prevent hypoxemia and hypercapnia, which could translate to improved anesthesia management.

Efficacy endpoints were assessed using a transcutaneous CO_2_ monitor (TCM). This device measured the changes in CO_2_ concentration during treatment. Transcutaneous CO_2_ (PtcCO_2)_ concentrations of 60 mm Hg or more (PaCO_2_ > 55 mm Hg) were considered marked hypercapnia. PtcCO_2_ concentrations of 50 to 60 mm Hg or more (equivalent to PaCO_2_ > 45 mm Hg) were considered moderate hypercapnia.

Furthermore, the incidence of hypoxemia with a transcutaneous oxygen saturation value of 90% or less, and whether the use of NHF was effective in preventing this adverse clinical event were evaluated.

**Discussion:**

The purpose of this study was to obtain evidence for the utility of NHF as a potential therapeutic device for patients undergoing an ERCP under sedation, assessed by determining if the incidence rates of hypercapnia and hypoxemia decreased in the NHF device group, compared to the control group that did not use this device.

**Trial registration:**

The study was registered in the jRCTs 072190021.

URL https://jrct.niph.go.jp/en-latest-detail/jRCTs072190021.

## 1 Introduction

For relatively invasive upper gastrointestinal endoscopy procedures, such as an endoscopic retrograde cholangiopancreatography (ERCP), and also lower gastrointestinal endoscopy procedures, anesthesia is routinely used to reduce patient anxiety. However, with the use of intravenous anesthesia, even at mild to moderate depth of anesthesia, there is always a risk of upper airway obstruction due to a relaxation of the upper airway muscles. “Guidelines on sedation in endoscopic practice” report on respiratory depression during an ERCP, and how it relates to sedation.^[1,2]^ It has been reported that the frequently used deep sedation method of 4 to 5 mg of midazolam and 70 to 100 mg of pethidine hydrochloride during an ERCP, is associated with the occurrence of respiratory depression at rates as high as 85%.^[3]^

Interestingly, it has been reported that respiratory complications that occur during intravenous sedation have a higher risk of hypercapnia than hypoxemia.^[1,4–7]^

Moreover, when low-flow oxygen is administered through a nasal cannula, the apparent percutaneous oxygen saturation value is maintained at a normal concentration; however, hypoventilation is sustained, which may result in impaired exhalation. The conditions of a gradual CO_2_ accumulation, and also CO_2_ concentrations maintained at 60 mm Hg or higher, are risk factors for secondary circulatory abnormalities such as an abnormal increase in blood pressure, tachycardia, and arrhythmia.

Furthermore, in recent years, the method of conventional air insufflation into the intestine during an ERCP, has been widely replaced by insufflating CO_2_ instead. It has been speculated that intestinal CO_2_ insufflation might reduce patient pain. However, it has been reported that CO_2_ in the intestinal tract is transiently absorbed into the bloodstream, and due to this observation, it is necessary to monitor patients for hypercapnia. In a study in which transcutaneous CO_2_ (PtcCO_2_) was measured during an ERCP,^[4]^ cases in which sedation was adjusted using PtcCO_2_ as an index during treatment were more likely to have PtcCO_2_ values exceeding 40 mm Hg.

Therefore, it is extremely important to monitor and prevent transient or continuous CO_2_ accumulation under intravenous sedation to maintain safe respiratory management.

A candidate to further improve respiratory management under intravenous anesthesia, is the use of a device called nasal high flow (NHF), which provides humidified air to flow to the nasal cavity at a high flow rate. NHF is typically used in patients with obstructive sleep apnea syndrome and respiratory disorders. This device has begun to be widely applied to the prevention of hypoxemia. It has a mild positive pressure load (several cmH_2_O) that improves respiratory function in part by improving CO_2_ wash out and reducing CO_2_ rebreathing.^[8–10]^ In addition, most recently, improved respiratory management using NHF is being studied during the propofol sedation.^[11]^ The hypothesis is that NHF use could prevent and resolve the onset of hypercapnia associated with transient upper airway obstruction during procedural sedation.

The purpose of this study was to maintain upper airway patency by applying NHF as a respiratory management method during intravenous anesthesia to patients undergoing ERCP performed under intravenous anesthesia. In addition, this study investigates whether improving respiratory management could prevent intraoperative hypercapnia and hypoxemia.

## 2 Methods/design

### 2.1 Study design

The present study was designed in accordance with the Standard Protocol Items: Recommendations for Interventional Trials and Consolidated Standard of Reporting Trials 2010 guidelines.^[12,13]^

This was an open-label, investigator-initiated, single center study on the efficacy of NHF use in patients undergoing ERCP performed under intravenous anesthesia. Treatment schedule and outcome measures are shown in Table 1.

**Table 1:**
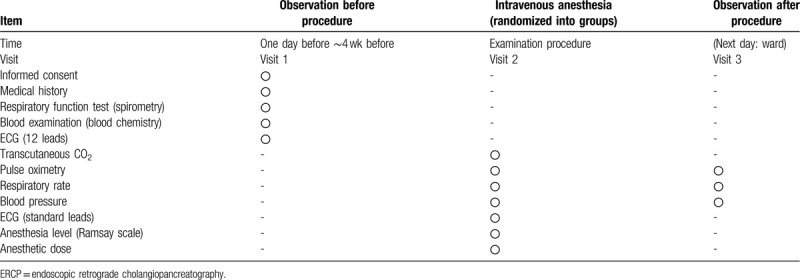
ERCP time schedule.

The Clinical Research Review Board in Nagasaki University approved the study and study protocol. The study was conducted at Nagasaki University Hospital in Japan. The study is registered on the jRCTs. The study was conducted in accordance with the principles of the Declaration of Helsinki and the established best clinical practices of Japan.

### 2.2 Participant recruitment

Participants were recruited from the Nagasaki University Hospital, where the subsequent study also took place. The treating clinical research coordinator (CRC) provided an explanation of the study to all participants, and these participants signed an informed consent form.

This was a randomized control study, comprised of 2 groups of participants. While both groups received intravenous anesthesia during ERCP, 1 group concomitantly used an NHF device.

After giving consent, subjects who met the registration requirements were randomly assignment to one of the 2 groups. Fifty-percent of patients are randomly assigned to each group.

### 2.3 Inclusion criteria

The following were inclusion criteria: adult patients between the ages of 20 and 85, that gave informed consent after a thorough explanation of all details of this clinical trial.

### 2.4 Exclusion criteria

The exclusion criteria were as follows:

1) continuous administration of oxygen by nasal cannula (home oxygen therapy),
2) inability to breathe through the nose,
3) use of antithrombotic drugs that could not be reduced or discontinued on the day before the endoscope,
4) a history of pneumothorax,
5) judged inappropriate as study subjects.

### 2.5 Study protocol

All participants underwent an ERCP under intravenous sedation, in a supine position on an examination table, in an endoscope room. Patients assigned to the NHF device group were cannulated with NHF in both nasal passages and provided with humidified air at a flow rate of 40 to 60 L/min.

A blood pressure monitor, electrocardiogram, and percutaneous oxygen saturation probe attached to the finger were used at the time of examination and treatment. Moreover, a transcutaneous CO_2_ probe was attached to the anterior chest or medial arm, and vital signs were continuously monitored. In order to continuously and non-invasively evaluate the circulating CO_2_ concentration, a transcutaneous CO_2_ concentration that was not affected even when inhaling high flow air with a nasal cannula was used. Transcutaneous CO_2_ was measured by a continuously attached electrode of the transcutaneous CO_2_ monitor (TCM) (Radiometer Inc., Japan) to the anterior chest and measuring the transcutaneous CO_2_ concentration together with the transcutaneous oxygen saturation and respiratory rate. Values were obtained from the anesthesia record of the electronic medical record (Nihon Kohden Inc., Japan). In the group receiving NHF, the values of vital signs 10 minutes after inhalation from the nasal cannula were recorded.

In the detection of the respiratory waveform, the respiratory phase, and the respiratory rate were evaluated based on the change in impedance between the electrodes using the three-electrode standard lead of the electrocardiogram.

To evaluate efficacy of NHF in the prevention of hypercapnia, the TCM output was used. PtcCO_2_ concentrations of 60 mm Hg or more (PaCO_2_ > 55 mm Hg) were considered marked hypercapnia. PtcCO_2_ concentrations of 50 to 60 mm Hg (equivalent to PaCO_2_ > 45 mm Hg) were considered moderate hypercapnia. Furthermore, the incidence of hypoxemia with a transcutaneous oxygen saturation value of 90% or less was evaluated. Results of these parameters determined whether the use of the NHF was effective in preventing the development of hypercapnia and hypoxemia.

Another parameter of the study that was assessed was the respiratory rate, evaluated by the change in impedance of an electrocardiographic (ECG) electrode. The depth of anesthesia was evaluated using the Ramsay scale, along with the total dose of the anesthetic and its dose per time.

In the NHF device group, patients used AIRVO2 (manufactured by Fisher & Paykel Healthcare) to inhale humidified air at a flow rate of 40 to 60 L/min from a nasal cannula.

### 2.6 Adverse events

In this study, the NHF device was secured to the patient via a nasal cannula, and this might have felt uncomfortable. Moreover, an electrode for transcutaneous carbon dioxide concentration measurements was attached to the anterior chest for both the NFH group and non-NHF group. For participants with sensitive skin, this might have caused temporary redness. When these adverse events occurred, care was taken to prevent undue harm to the research subjects.

### 2.7 Outcome

The primary endpoint was:

1) the occurrence rate of severe hypercapnia with a maximum transcutaneous CO_2_ concentration of 60 mm Hg or more (equivalent to PaCO_2_ > 55 mm Hg) during intravenous sedation,
2) area under the curve of percutaneous CO_2_ concentration per unit time during intravenous anesthesia (AUC),
3) duration of moderate hypercapnia showing maximum transcutaneous CO_2_ concentration of 50 mm Hg or more (equivalent to PaCO_2_ > 45 mm Hg) during intravenous sedation,
4) Occurrence rate of hypoxemia with percutaneous oxygen saturation value of 90% or less during intravenous sedation.

### 2.8 Efficacy

We evaluated the efficacy of the investigational device on a number of parameters, including: changes in CO_2_ concentration during treatment, the rate of occurrence of marked hypercapnia in which the maximum value of transcutaneous CO_2_ concentration is 60 mm Hg or more (equivalent to PaCO_2_ > 55 mm Hg), and the duration of moderate hypercapnia as defined by a transcutaneous CO_2_ concentration of 50 to 60 mm Hg (equivalent to PaCO_2_ > 45 mm Hg). Additionally, the incidence of hypoxemia was evaluated as defined by a transcutaneous oxygen saturation value of 90% or lower.

### 2.9 Safety

The safety evaluation indices of this clinical trial are as follows: adverse events are any undesired or unintended signs (including abnormal laboratory values, abnormal vital signs), symptoms, or illnesses that occur between the start of medical device (NHF) use and the end of the last observational study. This does not matter whether the study has a causal relationship. Symptoms and diseases occurring before the use of medical devices are treated as complications and not adverse events. However, if the complications worsen after the date of starting medical device use, they will be treated as adverse events, and the day on which the deterioration is confirmed will be the date of occurrence of the adverse events.

### 2.10 Data collection and management

The assignment table and input table used in this study were created with Research Electronic Data Capture (REDCap). The study was conducted after allocating the registered patients, and the data of all items in the medical record collected in the study were assigned to the researcher assigned the ID entered by physician, co-doctor and co-worker. The Principal Investigator or Co-Researcher approved the input observation/inspection/evaluation data of each research subject immediately after confirming the content.

For the data entered in the case report, the Principal Investigator and the Clinical Research Center Data Management staff perform a visual check and a logical check. Consequent to each check, if there are any problems or doubts in the data, the principal investigator, or the research coordinator is contacted. The case is fixed by performing data lock on the case when the issue has been resolved, and any modifications have been completed. If there is an error that needs to be corrected after the case is locked, the data management staff is responsible for overseeing this process.

In this study, monitoring will be carried out in accordance with the research plan and monitoring procedures to ensure that the research is being conducted properly.

### 2.11 Statistical analysis

Since this study is exploratory, we estimated an incidence rate for each study parameter and then used this information to calculate the necessary sample size for the verification study to achieve statistical significance.

Specifically, the rate of occurrence of hypercapnia in the NHF device group and the control group was calculated, and the difference (ratio) between the 2 groups and the 95% confidence interval on both sides were calculated. For other items, the average value and standard deviation for each group were manually calculated.

## 3 Discussion

The goal of using an NHF device, during an ERCP under intravenous anesthesia, was to prevent not only acute hypercapnia, but also hypoxemia. The clinical conditions resulting from a transient upper airway obstruction that can occur during intravenous anesthesia could be prevented or ameliorated by the use of an NHF device. It has been reported that the frequently used intravenous anesthesia during an ESD is associated with the higher occurrence rate of respiratory depression.^[3]^ Furthermore, it has been reported that respiratory complications that occur during intravenous anesthesia have a higher risk of hypercapnia than hypoxemia.^[1,4–7]^ Therefore, the prevention of hypercapnia could be important factor for achieving safe respiratory management during procedural sedation for ESD.

This is an exploratory study aimed at collecting information for conducting a verification study. Therefore, the number of cases is set based on the feasibility in our hospital. About 250 patients undergoing ERCP under intravenous anesthesia at Nagasaki University Hospital for 1 year. Approximately 125 cases, of which consent can be obtained from about 125 cases with a data collection period of 6 months, would be about 80 cases. The 80 ERCP cases are assigned to 2 groups, a device use group (40 cases) and a non-use group (40 cases), and stratified (20 cases each) by an assignment factor (COPD severity). Furthermore, the incidence of hypercapnia in the target population is estimated to be around 10% to 20%. For this reason, the number of events in each group was 40 cases, and the possibility of generating more than 1 event would be increased.

The primary endpoint of this study was:

1) the occurrence rate of severe hypercapnia with a maximum transcutaneous CO_2_ concentration of 60 mm Hg or more (equivalent to PaCO_2_ > 55 mm Hg) during intravenous sedation,
2) area under the curve of percutaneous CO_2_ concentration per unit time during intravenous anesthesia (AUC),
3) duration of moderate hypercapnia showing maximum transcutaneous CO_2_ concentration of 50 mm Hg or more (equivalent to PaCO_2_ > 45 mm Hg) during intravenous sedation,
4) occurrence rate of hypoxemia with percutaneous oxygen saturation value of 90% or less during intravenous sedation.

In addition, the evaluation of depth of anesthesia using Ramsay scale and total anesthetic dose during sedation will be also be assessed as a secondary endpoint.

Schumann et al reported that the availability to use NHF during sedation in an endoscopy suite reduced the requirement for anesthesia to perform complex endoscopic procedures.^[14]^ NHF reduces the rebreathing of expired CO_2_ from the anatomical dead space, which allows for maintained gas exchange at a lower minute ventilation.^[15]^ Therefore, patients can achieve the same alveolar ventilation with a reduced workload for the respiratory muscles.^[16]^

The most recent our study indicated that during sedation with propofol, NHF without supplemental oxygen attenuates CO_2_ retention and reduces the respiratory rate.^[11]^ The findings suggest that NHF can improve ventilation during procedural sedation for relatively invasive upper gastrointestinal endoscopy procedures, such as an endoscopic retrograde cholangiopancreatography, and also lower gastrointestinal endoscopy procedures, which may reduce the risk of complications related to hypoventilation.

## Acknowledgments

The authors would like to thank our colleagues and staff at the Dental Anesthesiology, Gastroenterology and Hepatology Department of Nagasaki University Hospital for their support.

## Author contributions

TA, TS, GM, SK, NH, and SN are responsible for conceiving and designing the trial, planning data analysis, drafting the manuscript, and approving the final manuscript. MP and ST are responsible for preparing and completing set up of AIRVO device including all equipment. EO, HS, NK, and KO will participate in data collection and are in charge of recruitment and treatment of patients. All authors will have access to the interim results as well as the capacity to discuss, revise, and approve the final manuscript.
